# Tissue Microbiome Associated With Human Diseases by Whole Transcriptome Sequencing and 16S Metagenomics

**DOI:** 10.3389/fgene.2021.585556

**Published:** 2021-03-04

**Authors:** Rana Salihoğlu, Tuğba Önal-Süzek

**Affiliations:** ^1^Bioinformatics Department, Graduate School of Natural and Applied Sciences, Muğla Sıtkı Koçman University, Muğla, Turkey; ^2^Computer Engineering Department, Faculty of Engineering, Muğla Sıtkı Koçman University, Muğla, Turkey

**Keywords:** neurodegenerative, vagina, tissue microbiome, whole transcriptome, RNA-seq, breast cancer, brain microbiome, 16S RNA analysis

## Abstract

In recent years, a substantial number of tissue microbiome studies have been published, mainly due to the recent improvements in the minimization of microbial contamination during whole transcriptome analysis. Another reason for this trend is due to the capability of next-generation sequencing (NGS) to detect microbiome composition even in low biomass samples. Several recent studies demonstrate a significant role for the tissue microbiome in the development and progression of cancer and other diseases. For example, the increase of the abundance of *Proteobacteria* in tumor tissues of the breast has been revealed by gene expression analysis. The link between human papillomavirus infection and cervical cancer has been known for some time, but the relationship between the microbiome and breast cancer (BC) is more novel. There are also recent attempts to investigate the possible link between the brain microbiome and the cognitive dysfunction caused by neurological diseases. Such studies pointing to the role of the brain microbiome in Huntington’s disease (HD) and Alzheimer’s disease (AD) suggest that microbial colonization is a risk factor. In this review, we aim to summarize the studies that associate the tissue microbiome, rather than gut microbiome, with cancer and other diseases using whole-transcriptome analysis, along with 16S rRNA analysis. After providing several case studies for each relationship, we will discuss the potential role of transcriptome analysis on the broader portrayal of the pathophysiology of the breast, brain, and vaginal microbiome.

## Introduction

The human body hosts a microbiome of microbes, bacteria, and viruses (Morgan and Huttenhower, 2012) that reside in human tissues and biofluids accompanied by various anatomical sites (e.g., mammary glands, placenta, and ovarian follicles; Marchesi and Ravel, 2015). The majority of the studies on the human microbiome are focused on microbial diversity and interactions only with the surface or the epithelial layer. Sequencing analyses of microbiomes have mostly focused on taxonomy profiling using 16S-rRNA amplicon sequencing, which efficiently covers the biodiversity of the samples using minimal sequencing by directly characterizing the microbiome taxonomy (Shakya et al., 2019). The whole-transcriptome analysis offers an alternative to 16S-rRNA sequencing by detecting and quantifying the low expression levels including non-coding RNAs (Shakya et al., 2019).

The link between the human microbiome and some specific diseases/cancer types has been tackled by several comprehensive reviews (e.g., vaginal microbiome, Ma et al., 2012; Muls et al., 2017; Champer et al., 2018; Xu et al., 2020; breast microbiome, Chadha et al., 2020; and brain microbiome, Piacentini et al., 2014; Harris and Harris, 2018).

In this review, rather than the commonly studied gut microbiome, we summarize the recent but less frequent microbiome studies analyzing the important role of the *whole tissue* microbiome not limited to but including the *epithelial* microbiome, providing a more wholesome picture of the association of dysbiosis with cancer, neurodegenerative, and inflammatory diseases.

Various omics technologies such as transcriptomics, proteomics, metabolomics, metagenomics, and their combinations provide new insights into the understanding of the human microbiome and its role in cancer/disease development (Komorowski and Pezo, 2020). As most of the microorganisms can not be practically cloned and cultured by the conventional methods, most of the recent microbiome studies utilize 16S/18S/ITS Amplicon sequencing or whole transcriptome analysis. Among these techniques, our mini-review focuses on 16S-rRNA and whole transcriptome analysis; both of which are demonstrated to be equally sensitive in bacterial genus detection (Razzauti et al., 2015). Of 16S-rRNA and whole transcriptome analysis, whole transcriptome analysis is more robust and cost-effective in both capturing the coding and non-coding RNA and quantifying the heterogeneity in gene expressions of cells, tissues, and organs (Ozsolak and Milos, 2011; Sancesario and Bernardini, 2018). The advantages of the whole transcriptome analysis compared to other omic methods are vast, the most fundamental one being its contribution to the determination of new strategies for drug discoveries and therapeutic interventions (Ozsolak and Milos, 2011; Jiang et al., 2015).

This mini-review aims to provide a summary of the recent studies related to the role of the issue microbiome changes in cancer/unhealthy tissues of the brain, breast, and vagina using 16S-RNA and whole transcriptome analysis. The studies considering the tissue microbiome as originating not only from the surface but all parts of the aforementioned tissues are included within the scope of this article.

## Breast Intracellular Microbiome

**FIGURE 1:**
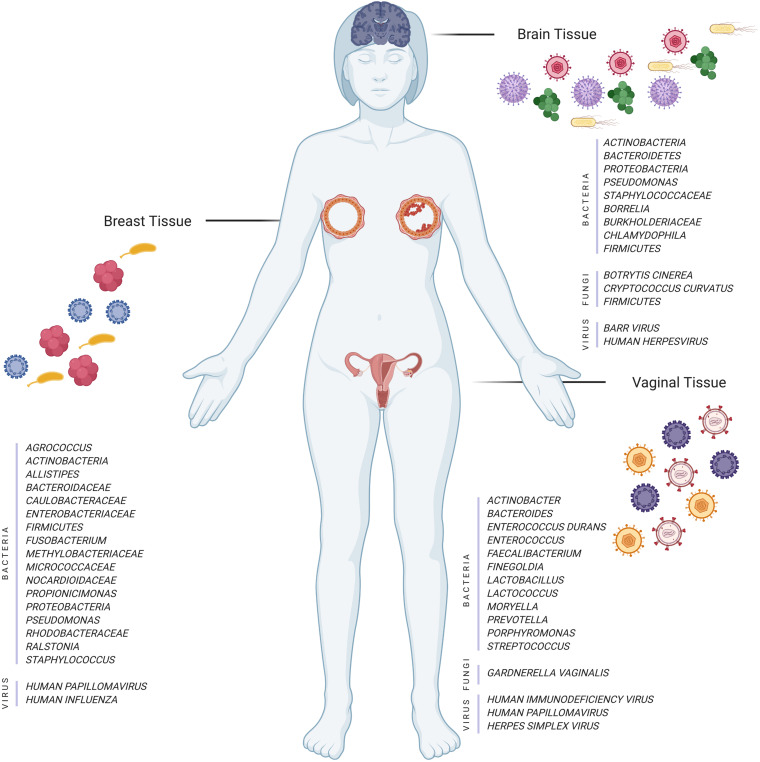
Illustration of different tissues and their microorganisms (bacteria, fungi, and viruses) in normal and disease cases (Created with BioRender.com).

Breast cancer (BC) microbiome (Figure 1) is hypothesized to be affected by bacteria-related inflammation in the mammary ducts and glands disrupting the hierarchy of stem cells. Several recent studies focus on the intra-tissue microbial and the viral composition in BC [e.g., *human papillomavirus* (HPV); Nejman et al., 2020; Sher et al., 2020]. HPVs are more abundant in BCs compared to benign breast and normal breast controls (Lawson et al., 2016). Some studies claim that HPV may play a role in the formation of breast ductal carcinoma, together with the ability to immortalize human epithelial cells (Di Lonardo et al., 1992; Kan et al., 2005; Heng et al., 2009). In addition to the epithelial tissue microbiome studies, several recent studies point to the presence of intra-tissue bacteria in healthy controls (Nejman et al., 2020).

Some studies have shown that pregnancy and breastfeeding might reduce the risk of BC due to the protective behavior of the lactose fermenting bacterial flora in the mammary ducts (Marwaha et al., 2020). Another important finding related to BC is that some patients with hormone receptor-positive (HR+) tend to have more aggressive BC possibly due to the dysbiosis that triggers the early inflammation in the mammary gland during the progression of HR+ breast tumor by disrupting the mammary tissue homeostasis (Rosean et al., 2019).

The presence of mucosal-associated invariant T (MAIT) cells in the breast ducts intervenes T-helper 17 cell responses that might be regulated during breast carcinogenesis by the indications of breast microbiome and the expression of stress-related ligands by neoplastic breast duct epithelial cells (Zumwalde et al., 2018).

Live and metabolically active *Proteobacteria*, *Firmicutes*, and *Actinobacteria* are discovered in breast tumors (Nejman et al., 2020). Thompson et al. (2017) pointed to the increase of *Proteobacteria* in tumor tissues and *Actinobacteria* in normal tissues. Additionally, the correlation of expression profiles with the microbiome data indicates that *H. influenza* is significantly correlated with genes in the G2M checkpoint, E2F transcription, and mitotic spindle assembly pathways (Thompson et al., 2017) (Table 1).

**TABLE 1:** Summary of recent findings on the breast, brain and vagina tissue microbiome.

**Tissue**	**Sample type and size**	**Methods**	**Finding**	**References**
Breast	13 fresh breast tissue benign or 45 cancerous tumors, and 23 controls	16S-rRNA	*Enterobacteriaceae*, *Bacillus*, *Staphylococcus* species are abundant in breast cancer.	Urbaniak et al., 2016
Breast	Breast tumor tissues (668) and non-cancerous adjacent tissues (72) from Cancer Genomics Hub (CGHuB)	16S-rRNA	*Proteobacteria* showed abundant shifts in tumor samples. It appears that *H. influenza* strain is associated with genes in the G2M control point, E2F transcription, and mitotic spindle assembly pathways, and *L. fleischmannii* strain is associated with genes including epithelial to mesenchymal transition. The relationship between *S. pyogenes* abundance and GUSBP4, GUSBP9, and GPA2 expression levels shows the role of this species in the exposure of the local breast environment to higher estrogen levels.	Thompson et al., 2017
Breast	50 endocrine receptor (BRER) positive, 34 human epidermal growth factor receptor 2 (HER2) positive (BRHR), 24 triple positive (BRTP), and 40 triple-negative (BRTN) breast cancer tissues plus 20 healthy controls	Whole-genome and transcriptome	**BRER:** *Bifidobacterium, Arcanobacterium, Citrobacter, Cardiobacterium, Escherichia, Filobasidilla, Brugia, Mucor, Trichophyton, Paragonimus* **BRHR:** *Streptococcus, Nodaviridae, Epidermophyton, Fonsecaea, Pseudallescheria, Balamuthia* **BRTP:** *Trichostrongylus, Chlamydophila, Hepeviridae, Bordetella, Campylobacter, Chlamydia, Birnaviridae, Legionella, Pasteurella, Penicillium, Ancylostoma, Angiostrongylus, Echinococcus, Trichomonas, Sarcocystis*, **BRTN:** *Geobacillus, Orientia, Rothia, Arcobacter, Alternaria, Malassezia, Aerococcus, Rhizomucor, Piedraia, Centrocestus, Trichuris, Contracaecum, Leishmania, Necator, Toxocara Onchocerca, Trichinella*.	Banerjee et al., 2018
Breast	22 benign and 72 malignant breast cancer samples	16S-rRNA	*Micrococcaceae*, *Caulobacteraceae, Propionicimonas, Rhodobacteraceae, Nocardioidaceae*, and *Methylobacteriaceae* are abundant in malignant disease. *Methylobacteriaceae* family is the only common biomarker/signature in the tissue microbiomes between tumor/malignant and normal/benign.	Meng et al., 2018
Breast	19 breast tissues (non-Hispanic Black women –NHB), 62 total breast samples (non-Hispanic White women-NHW); 11 donors from breast cancer and adjacent normal breast tissue	16S-rRNA	*Proteobacteria* is the most abundant in normal, normal adjacent to tumor, and breast tumors from NHB and NHW women, with fewer *Firmicutes*, *Bacteroidetes*, and *Actinobacteria*. The racial difference is reported in breast tissue microbiome (e.g., higher abundance of genus *Ralstonia* in NHB women compared to NHW tumors).	Smith et al., 2019
Breast	15 breast tumor tissue from women who underwent neoadjuvant chemotherapy, 18 women with no prior therapy at the time of surgery	16S-rRNA	The chemotherapy administration increases the breast tumor *Pseudomonas* spp. Treatment of breast cancer cells with *Pseudomonas aeruginosa* conditioned media differentially effected proliferation in a dose-dependent manner and modulated doxorubicin-mediated cell death.	Chiba et al., 2020
Brain	All samples from a brain bank	Internal Transcribed Spacer (ITS)	*Botrytis cinerea and Cryptococcus curvatus* are common to all four central nervous system regions. Five genera are common to all nine AD patients: *Alternaria, Botrytis, Candida, Cladosporium*, and *Malassezia*.	Alonso et al., 2017
Brain	Consortium to Establish a Registry for AD (CERAD) criteria and Braak stage on 24 AD and 18 age-matched controls.	DNA sequences	Lipopolysaccharide and *E. coli K99* are greater in AD compared to control brains. Gram-negative bacterial molecules are related to AD neuropathology.	Zhan et al., 2016
Brain	Control = 12, AD = 14	16S-rRNA	The control brain has similar bacterial profiles with the blood, but the AD brain displays a larger proportion of *Actinobacteria*.	Emery et al., 2017
Brain	Accelerating Medicines Partnership AD; Knowledge Portal	16S-rRNA	The increase in the abundance of HHV-6A, HHV-7, and herpes simplex virus (HSV-1) genomes in banked postmortem brains from subjects with AD compared to controls.	Readhead et al., 2019
Brain	Postmortem hippocampal formation specimens from 10 neurological control and 10 AD cases, 22 AD specimens, and 19 neurological controls from the hippocampus; 12 control and 20 AD cerebellum samples	16S-rRNA	The most abundant phyla in both cancer and normal cases are *Proteobacteria*, *Firmicutes*, *Actinobacteria*, and *Bacteroidetes*. There is a significant role of brain region on the presence of microbial DNA revealed by the variations in beta diversity in hippocampal and cerebellum samples.	Westfall et al., 2020
Brain	Examination of the olfactory bulbs from autopsy of two AD cases	Bacterial DNA	The HSV1 is a strong risk factor when it is present in the brain of carriers of the type 4 allele of the gene for APOE-ε4. Another important risk factor is the bacterium, *Chlamydia pneumoniae* identified and localized in the AD brain.	Itzhaki et al., 2004
Brain	Samples of tissue from brain donors, AD, and control individuals	16S-rRNA	No evidence for expression of early (ICP0) or late (ICP5) proteins of the HSV-1 in brain. A polyclonal antibody against *Borrelia* detected structures that appeared not related to spirochetes, but rather to fungi, revealing the presence of several bacteria in brain tissue of AD patients.	Pisa et al., 2017
Brain	118 human viruses, and PCR from 711 AD and non-AD control brains	Whole transcriptome	There is no clear relation between HHV-6 and AD, whereas the Epstein–Barr virus (EBV) and cytomegalovirus (CMV) are comparable.	Allnutt et al., 2020
Vagina	151 healthy women (65 HPV+ and 86 HPV−)	16S rDNA	*Bacteroides plebeius*, *Acinetobacter lwoffii*, and *Prevotella buccae* are significantly seen in HPV-positive women.	Chao et al., 2019
Vagina	Women with the age of 18–29 who have applied to the sexually transmitted infections (STIs) clinic have symptoms	Whole transcriptome	The samples from women with STI infection only contain pathogen-specific sequences (3–38% transcriptome coverage).	O’Connell et al., 2019

Although the microbiome profiles of malignant and benign breast tumors are different (Costantini et al., 2018; Meng et al., 2018), there are some significant similarities in the profiles of normal and tumor breast tissues revealed by 16S rRNA amplicon sequencing (Urbaniak et al., 2016) (Table 1). The genus *Propionicimonas* and families *Micrococcaceae*, *Caulobacteraceae*, *Rhodobacteraceae*, *Nocardioidaceae*, and *Methylobacteriaceae* in malignant tissues are the enriched microbial biomarkers, and the development of malignancy results in the decrease in *Bacteroidaceae* and the increase in *Agrococcus* (Meng et al., 2018) (Table 1). Additionally, the genus *Fusobacterium*, *Bacteroides*, and *Allistipes* are especially related to BC (Philley et al., 2019.). Another important finding is the racial differences in the microbiome of breast tissue first identified by Smith et al. (2019), i.e., relatively higher abundance of the genera Ralstonia in non-Hispanic Black women (Table 1).

The effect of HPV on BC has been investigated for years (Di Lonardo et al., 1992; Widschwendter et al., 2004; Sher et al., 2020). The presence of HPV in BC tissue has resulted in increased histopathological activity in the tumor (Al-Badry et al., 2019). This situation may also be presented with the pathologic nipple discharge (PND; Balcı et al., 2019). Also, the serum derived-extracellular vesicles (EVs) from the BC patients containing HPV DNA reveal the role of HPV as a potential trigger for aggressive BC (De Carolis et al., 2019). The expressions of *P53*, *RB*, *BRCA1*, and *BRCA2* in HPV positive BC patients are reduced compared to those in HPV negative ones, possibly indicating the positive relationship between the increase in the inflammatory cytokines (e.g., IL-1 and IL-6) and tumor progression (Khodabandehlou et al., 2019).

Chemotherapy, unfortunately, has significantly exacerbated the disease progression by shifting the microbiome of breast tumors and increasing the *Pseudomonas* spp. (Chiba et al., 2020) (Table 1). There is a need for further studies that cover a larger and more racially diverse data population, and any findings will provide new insights into the role of the microbiome in the therapy stage and the investigation of novel bacterial biomarkers (Chiba et al., 2020). In summary, the recent developments related to the role of the microbiome in BC reveal the importance of this relationship for the investigation of BC (Sher et al., 2020).

## Brain Microbiome

An interesting indication of tissue microbiome is its association with neurodegenerative diseases. The polymicrobial infections are comprised of fungi and bacteria found in the brain tissues of AD patients (Pisa et al., 2015, 2017). Many other studies claimed that the repeated activation of herpes simplex virus 1 (HSV-1) promotes the neurodegeneration aspect of AD (Deatly et al., 1990; Mori et al., 2004; Mancuso et al., 2014). Microbial colonization is also considered as a possible risk factor for Huntington’s disease (HD) (Alonso et al., 2019). Alzheimer’s disease (AD) is a neurodegenerative disorder whose pathogenesis is not only limited to the neuronal compartment but also accompanied by a significant interaction with the immunological mechanisms in the brain (Heneka et al., 2015). Even though the etiopathogenesis of AD has not been well documented, some investigations point to the role of the microbiome (Figure 1) (gut, Vogt et al., 2017; oral, Shoemark and Allen, 2015), and the etiology of AD has been considered as microbial (Bhattacharjee and Lukiw, 2013; Pisa et al., 2017).

The role of human herpesvirus (HHV) in the etiology of AD has been evident recently, yet studies on the different viruses of the herpes family [e.g., HHV-6, -7, cytomegalovirus (CMV), Epstein–Barr virus (EBV)] are limited (Carbone et al., 2014). Nevertheless, there are some recent additional attempts to reveal the relation between HHV and AD (Eimer et al., 2018; Readhead et al., 2018, 2019). For instance, HHV-6A and 7 have been detected in AD patients with a higher viral abundance of amyloid precursor protein (APP) (O’Brien and Wong, 2011) metabolism (i.e., induction of APBB2, APPBP2, BIN1, BACE1, and CLU) (Readhead et al., 2018). The oligomers of Amyloid-β (Aβ) peptide bind HHV surface glycoproteins and accelerate the deposition of β-amyloid (Eimer et al., 2018). This leads to protective viral entrapment activity against neurotropic HSV-1 and also HHV6A-B that are linked to AD (Eimer et al., 2018). Besides, the increase of the KIR2DL2/C1 genotype in AD patients and the lower anti-herpetic activity of KIR2DL2 positive natural killer (NK) cells support the role of HHV infection in AD development and also increase the susceptibility to HHV-6A infection (Rizzo et al., 2019). Most studies also suggest the prevalence of HHV-6A in the AD brain compared to others (Eimer et al., 2018; Readhead et al., 2019; Rizzo et al., 2019). Besides, the effect of HSV-1 in the development of AD among people with the genetic susceptibility factor of the apolipoprotein E (APOE4) allele has been evident (Lindman et al., 2019; Linard et al., 2020).

The contribution of infectious microbial components and also virulence factors rhamnolipids (RLs) to the pathophysiology of the human central nervous system (CNS), including AD, has been potentially important (Andreadou et al., 2017). The presence of RLs is attributed to chronic bacterial infections forming bacterial virulence factors secreted by a wide variety of pathogens (Andreadou et al., 2017). The fungi in the samples of the frontal cortex in AD brains are distinct, indicating the varying microbial compositions among brain regions (Alonso et al., 2018; Westfall et al., 2020) (Table 1). A polyclonal antibody against *Borrelia* has been identified in structures associated with fungi (Pisa et al., 2017). Two independent *Chlamydophila* antibodies have revealed several structures similar to fungal cells and hyphae and prokaryotic cells, but are most likely unrelated to *Chlamydophila* spp. Several bacteria in the AD patients suggested that the polymicrobial infections are comprised of fungi and bacteria occurred in their brain tissues (Pisa et al., 2017) (Table 1). The fungal species found in the CNS of AD patients have been investigated by next-generation sequencing (NGS) that revealed the most common species, namely, *Botrytis cinerea* and *Cryptococcus curvatus* (Alonso et al., 2017). *Burkholderiaceae* and *Staphylococcaceae* are more abundant in AD brains compared to normal brains (Alonso et al., 2018). Studies on that subject aiming at investigating the link between fungal species with the AD are rather essential for providing antifungal therapy and also for the evolution and severity stages of clinical symptoms in AD patients (Alonso et al., 2017) (Table 1). On the other hand, microbiological attack or change is thought to be one of the factors causing CNS disorders, also evident for the AD that shows an increase in bacterial populations (e.g., large amount of *Actinobacteria*; Emery et al., 2017) (Table 1).

Huntington’s disease is caused by a triplet expansion in the Huntingtin (HTT) gene (Vonsattel and Difiglia, 1998). Alonso et al. (2019) first identified the role of some prevalent bacterial genera (*Pseudomonas*, *Acinetobacter*, and *Burkholderia*) in the brain microbiome of HD patients. RNA-seq analysis of human neurodegenerative disease tissues (except for AD) reported no significant difference compared to cytotoxic T-lymphocytes (CTL) tissues, indicating that the sub-clinical infections do not result in the inflammation related to the tissue of many neurodegenerative diseases such as amyotrophic lateral sclerosis (ALS) and Parkinson’s disease (PD) (Bennett et al., 2019).

## Vaginal Microbiome

The relation between the vaginal microbiome (Figure 1) and the high-risk HPV infection has been propounded by several studies (Chao et al., 2019, 2020; Keller et al., 2019; Liu et al., 2019; Nené et al., 2019; Zhou et al., 2019; Abudula et al., 2020; Jiang et al., 2020). The sequencing of 16S rRNA genes reveals that some anaerobic bacteria (e.g., *Bacteroides plebeius* and *Acinetobacter lwoffii*) are significantly more common in HPV positive women, suggesting a specific microbiome as a biomarker to detect changes in the cervical microenvironment indicating HPV infection (Chao et al., 2019). The genus *Prevotella*, *Porphyromonas*, and *Enterococcus* are the highest in the cervical permanent HPV infection, whereas the *Bacteroides* genus is the lowest (Chao et al., 2020).

The expression studies on cervical lesions to explore the possible relation of HPV with cervical cancer are also crucial. Toll-like receptor 4 (TLR4) expression supports the claim of a distinct relation between tumor formation and HPV-positive cervical cancer (Jiang et al., 2020), suggesting that TLR4 somehow enables the formation of a local immunosuppressive microenvironment.

*Human papillomavirus*, human immunodeficiency virus (HIV), and HSV have been associated with the growth of genital-related cancers. Keller et al. (2019) proclaim that the increase in microbial diversity and cervicovaginal inflammation in women with HIV+ and HSV+ significantly perturbs genital health. It is thought that neither the inception of antiretroviral therapy (ART) nor the restructuring of the immune system affects the vagina microbiome of HIV-infected women (Liu et al., 2019). Several 16S rRNA sequencing studies (Nené et al., 2019; Zhou et al., 2019) demonstrate a decrease in bacterial diversity in ovarian cancer tissues compared to normal ones. Some bacterial implications are suggested as biomarkers for the early detection of ovarian tumors, such as higher ratios of two phyla for *Proteobacteria*/*Firmicutes*, and the increase of genus *Acinetobacter* and decrease of genus *Lactococcus* (Zhou et al., 2019).

## Discussion and Conclusion

In this mini-review, we mainly discuss the possible effects of human tissue microbiome in the development of some common cancers and neurodegenerative diseases. There might be two important implications of our comprehensive literature compilation: the first implication being the presentation of the limited number of studies that deal with the microbiome differences in healthy and unhealthy/cancer tissues, for example, AD is more commonly studied for its relationship with the oral and gastrointestinal microbiome. The second implication of our literature compilation is to list the shared bacteria (e.g., *Proteobacteria*, *Bacteroides*, and *Firmicutes*) that display either positive or negative anomalies common to all cancer tissues discussed above (Figure 1).

Many of the recent findings summarized in Table 1 are now possible due to the ability of the whole transcriptome analysis to (1) provide abundance information along with the taxonomic diversity and (2) provide a vaster picture of the expression profile of the microbiome comprising of fungal, viral, and bacterial along with host expression profile. In some of the cases summarized in Table 1, observed differences in each patient are due to fungal species which could not be discovered if a whole transcriptomic approach is not taken (Alonso et al., 2017, 2019).

Variations in the microbiome of cancer patients with different cancer stages have already been known in the literature. For instance, the species of *Firmicutes* and *Bacteroides* are dominant in the invasive and benign BC, whereas some of the species such as *Fusobacterium*, *Atopobium*, and *Lactobacillus* are enriched in malignant breast tissues (e.g., Hieken et al., 2016; Chadha et al., 2020). The recent studies about the role of the microbiome in different cancers and neurodegenerative diseases reveal that there are some common species observed in all cancer tissues. For instance, some of these species (e.g., *Bacteroides*) are enriched in the AD tissues, while they are not abundant in cervical and BC tissues. Additionally, *Proteobacteria* and *Firmicutes* are enriched in all types of cancer tissues. Such similarities and/or differences may be attributed to differences in tissue structure. Our mini-review will attract more attention to the reprocessing of the publicly available RNA-seq data to distinguish microbial contamination from tissue microbiome via *in silico* techniques to clarify the relative composition, abundance, and impact of microbiome in cancer tissues.

Most of the studies on the possible link between microbiome and diseases associating the presence of many potential microbial biomarkers and their pathways with the advanced stage of the diseases offer new insights into the diagnostic staging (e.g., Wozniak et al., 2005; Alonso et al., 2017; Meng et al., 2018; Rizzo et al., 2019). There is also some evidence that the profiles of microbiome in healthy and unhealthy tissues (e.g., for BC; Urbaniak et al., 2016) are identical, and hence the role of tissue microbiome for the development of diseases/cancers should be further investigated by increasing the number of available studies. As the Next-Generation Sequence (NGS) technology becomes more precise and novel *in silico* techniques are getting developed to discard the studies with bacterial contamination and non-standardized analysis pipelines, the re-analysis of the existing transcriptome data offers a huge potential for the future *in silico* data-mining microbiome studies from the vast amount of publicly available transcriptomic data.

## Author Contributions

RS wrote the manuscript and TÖS supervised the project. Both authors discussed the results and contributed to the final manuscript.

## Conflict of Interest

The authors declare that the research was conducted in the absence of any commercial or financial relationships that could be construed as a potential conflict of interest.
